# A Rare Case of Neuroendocrine Tumor in a Patient With Neurofibromatosis Type 1: Is There Any Association?

**DOI:** 10.7759/cureus.29621

**Published:** 2022-09-26

**Authors:** Fatima Zahra Baddi, Fatima Zohra Ahsayen, Hiba Ramdani, Meriem Rhazari, Imane Skiker, Afaf Thouil, Hatim Kouismi

**Affiliations:** 1 Department of Respiratory Diseases, Mohammed VI University Hospital, Oujda, MAR; 2 Department of Pulmonology, Faculty of Medicine and Pharmacy, Mohammed I University, Oujda, MAR; 3 Department of Radiology, Mohammed VI University Hospital, Oujda, MAR; 4 Department of Radiology, Faculty of Medicine and Pharmacy, Mohammed I University, Oujda, MAR; 5 Departement of Medicine, Mohammed VI University Hospital, Oujda, MAR; 6 Department of Medicine, Faculty of Medicine and Pharmacy, Mohammed I University, Oujda, MAR

**Keywords:** neurofibromatosis type 1 (nf1), risk factor, lung cancer, malignancies, neuroendocrine tumor

## Abstract

Neurofibromatosis type 1 (NF1) is an autosomal dominant condition characterized by café-au-lait spots, cutaneous neurofibromas, axillary and inguinal freckling, and iris Lisch nodules; however, the presentations vary greatly, even within families. NF1 is also a recognized risk factor for the development of malignancy particularly malignant peripheral nerve sheath tumors (MPNST), optic gliomas, other gliomas, and leukemia. Nevertheless, the occurrence of lung cancer in a patient with neurofibromatosis type 1 is a rare phenomenon.

Here we present a case of neuroendocrine tumor in a patient with neurofibromatosis type 1, highlighting the association between the two diseases. This case report also aimed to raise awareness of possible malignancies in patients with neurofibromatosis type 1.

## Introduction

Neurofibromatosis type 1 (NF1), formerly known as von Recklinghausen's disease, is an autosomal dominant disease that is one of the most prevalent genetic disorders [1] with an estimated prevalence of one in 3000 births [2]. NF1 is induced by loss-of-function mutations in the NF1 gene, located on chromosome 17q11.2, encoding neurofibromin 1. Neurofibromin 1 is a protein related to tumor suppression [3]. Skin pigmented lesions known as "café-au-lait spots," axillary freckling, and cutaneous neurofibromas are all symptoms that can be observed clinically in NF1. NF1 is a risk factor for malignancy such as malignant peripheral nerve sheath tumors and gliomas and leukemia [1]. However, the occurrence of neuroendocrine tumor of lung is rare.

According to the World Health Organization, lung neuroendocrine tumors are classified into the following four histologic subtypes: carcinoids (typical {TC} and atypical {AC}), large-cell neuroendocrine carcinoma (LCNEC), and small-cell lung carcinoma (SCLC) (WHO) [4].

In light of this, we report a case of a 37-year-old female with neurofibromatosis type 1, followed since the age of 12 years, who developed a neuroendocrine tumor of the lung. The relation between NF1 and neuroendocrine tumor of lung is discussed.

## Case presentation

**Figure 1:**
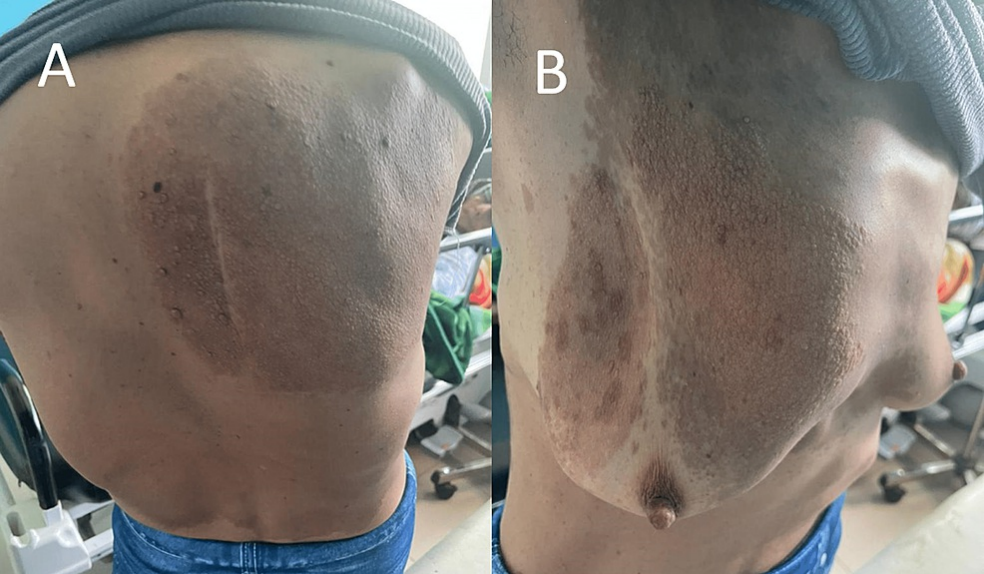
Clinical aspect of café-au-lait spots with neurofibromas at the back (A) and the trunk (B) of the patient.

A 37-year-old female, lifetime non-smoker with congenital kyphoscoliosis is followed since the age of 12 years for neurofibromatosis type 1 (NF1) with no other personal or familial history. The patient reports a 10-year history of chronic dyspnea grade I. She was admitted to the pulmonology department at our hospital after her condition started worsening two months ago with the progression of her dyspnea to grade III associated with a dry cough and xerostomia. At her first clinical presentation, she was apyretic (37.2°C). In the clinical examination, kyphoscoliosis with pectus carinatum was noted with multiple pigmented lesions of the skin called café-au-lait spots with neurofibromas all over the trunk and the back (Figures 1A, 1B).

**Figure 2:**
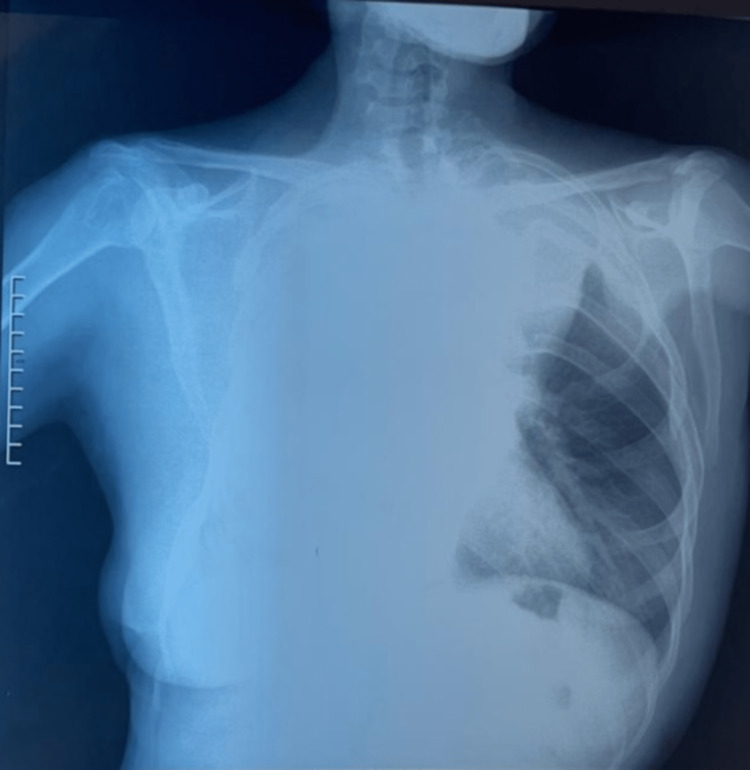
Chest x-rays of the patient. The image shows homogeneous fluid tone opacity in the upper 2/3 of right hemithorax associated with a heterogenous opacity in the lower 1/3 of the same side with pulmonary hyper-transparency and widening of the intercostal spaces.

The pulmonary examination found decreased vocal fremitus and breath sound in the right lung with dullness to percussion, while the examination of the left lung was normal. Other than that, our patient was conscious and alert, following all the commands, with a well-preserved motricity at four limbs, she was normotensive with blood pressure of 110/62 mmHg, heart rate of 75 beats per minute, respiratory rate of 18 breaths per minute, and had oxygen saturation of 95% on room air. The rest of the physical examination revealed no abnormalities. A chest x-ray was performed and revealed homogeneous fluid tone opacity in the upper 2/3 of right hemithorax associated with a heterogenous opacity in the lower 1/3 of the same side with pulmonary hyper-transparency and widening of the intercostal spaces (Figure 2).

**Figure 3:**
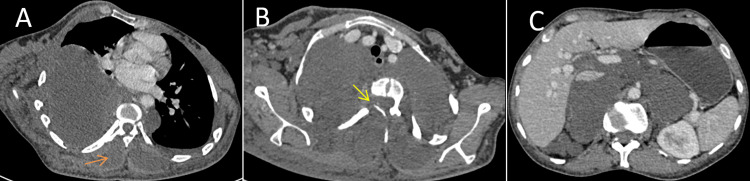
Post-contrast thoracoabdominal CT scan. CT scan, (A-C) axial sections, showing hypodense paravertebral masses, mostly necrotic with extension through the intervertebral foramen into the spinal canal next to T3 (yellow arrow), compressing lung and surrounding the abdominal aorta, associated with multiple subcutaneous and muscular lesions (orange arrow).

Due to highly suspected malignancy, a CT scan of the thorax and abdomen was obtained and showed hypodense paravertebral masses, mostly necrotic with extension through the intervertebral foramen into the spinal canal next to T3 compressing lung and surrounding the abdominal aorta, associated with multiple subcutaneous and muscular lesions (Figures 3A-3C).

**Figure 4:**
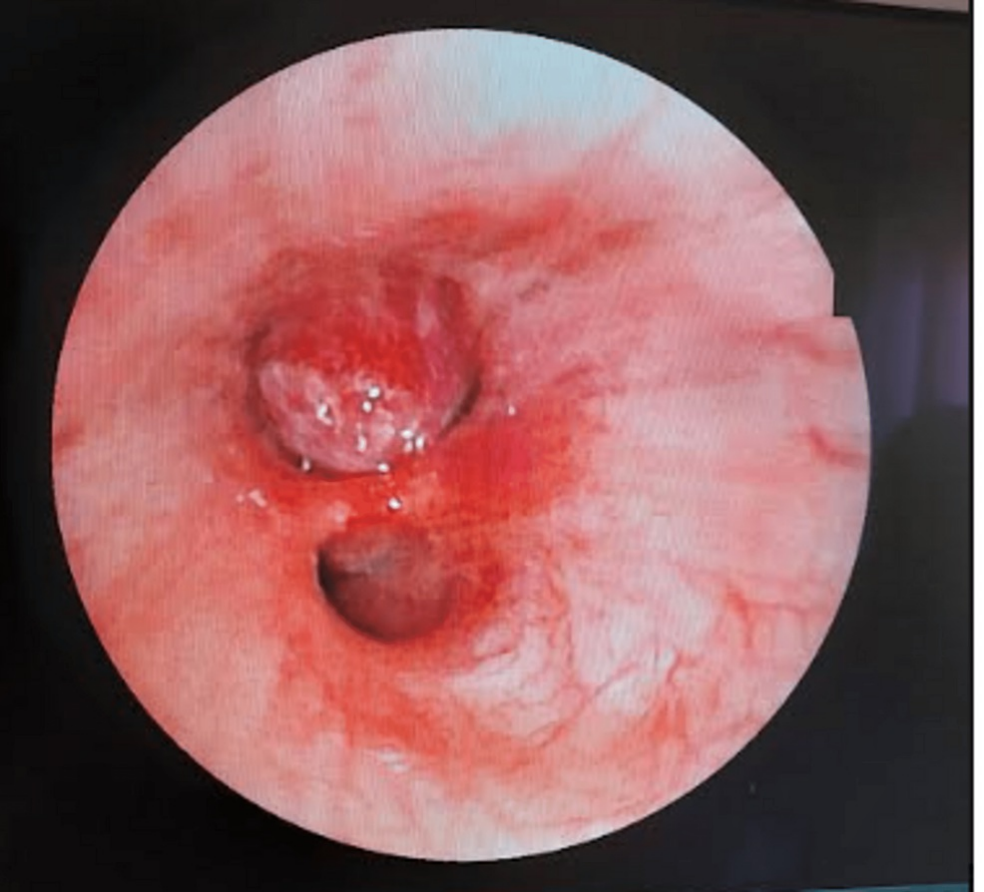
Bronchoscopic appearance showed a bud in the right bronchial tree completely obstructing the lower lobe.

**Figure 5:**
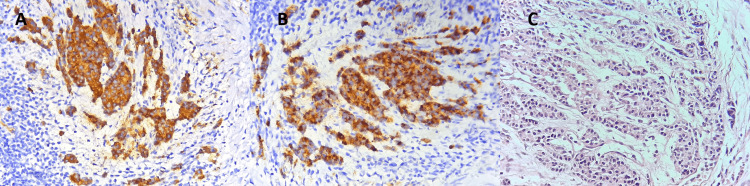
Histological examination of bronchial biopsies. The images show a proliferation of tumor cells arranged in organoid pattern consisting of nests and trabeculae of tumor cells separated by thin fibrovascular septa. Those cells are monotonous showing mild atypia; the nuclei are round with salt and pepper chromatin, and moderate eosinophilic cytoplasm. Immunohistochemical staining shows positivity of tumor cells for both synaptophysin (A) and chromogranin (B). No mitotic figures were observed (C).

A bronchoscopy with biopsy was performed and showed a bud in the right bronchial tree completely obstructing the lower lobe (Figure 4) which was biopsied (Figures 5A-5C) leading to the diagnosis of neuroendocrine tumor. The patient's case was reviewed by the thoracic surgery team and the decision was not to for the operative treatment. The patient was then referred to the oncology department for further treatment.

## Discussion

As mentioned earlier, NF1 is a risk factor for the occurrence of certain malignancies including lung cancers. NF1 increases the sensitivity of the lungs to cigarette smoking which in turn increases the risk of interstitial lung disease which further raises risk of cancer development [5]. However, the association of lung cancers with NF1 is uncommon and rarely reported in the medical literature [6].

To explain the link between NF1 and lung cancer, two basic hypotheses have been proposed. The first hypothesis is that since NF1 is a potential risk in the occurrence of interstitial lung diseases, the scars or bullae secondary to the latter can give way to the development of tumors [6,7]. The second hypothesis is the inactivation of the p53 tumor suppressor gene secondary to the deletion of chromosome 17p. Inactivation of p53 has been linked to the development of small-cell lung cancer in individuals with NF1 as well as the p53 mutation was detected in half of the patients with non-small-cell lung cancer [6]. This underscores that NF1 is a potential risk factor for lung cancer in non-smoking patients.

The association of pulmonary neuroendocrine tumors with NF1 is rarely reported in the medical literature. To the best of our knowledge, only one case has been reported in the literature concerning a 45-year-old patient with NF1 and active smoking [7]. Lung neuroendocrine tumors are a rare type of tumor originating in neuroendocrine cells with amine precursor uptake and decarboxylation (APUD) derived from Kulchitsky cells [8]. Pulmonary carcinoids are common in the fourth and sixth decades of life with a median age of 45 years [8]. In addition, smoking is not considered a risk factor unlike SCLC and LCNEC [9]. As our case illustrates, the patient had neither active nor passive smoking. The typical carcinoid is 10 times more frequent than the atypical carcinoid which is characterized by high metastatic potential in 50% of cases [10].

The clinical presentation of pulmonary neuroendocrine tumors is variable depending on the location, type, and size of each tumor. Nevertheless, the most frequent clinical manifestations in the case of carcinoid tumors are dyspnea, cough, chronic respiratory infections, and hemoptysis [11]. Our patient presented to our department with progressive dyspnea which worsened two months earlier, associated with dry cough and xerostomia. In the case of peripheral pulmonary carcinoid, the clinical picture is generally asymptomatic with most often accidental discovery [11]. Although not common, paraneoplastic syndromes can appear. For example, carcinoid syndrome which is considered characteristic is more common in gastrointestinal tumors and constitutes only 1-3% of tumors of pulmonary origin [12].

With routine chest x-rays, more than 40% of lung neuroendocrine tumor (LNET) cases are discovered incidentally [13]. Chest CT with contrast remains the gold standard. The most common radiographic findings were small, spherical pulmonary cysts and asymmetric, thin-walled bullae with apical prominence. In addition, bullae and cysts are thicker and more circumscribed in patients with NF1 [7]. For the identification of distant metastases, somatostatin receptor PET is of great help [14]. Because most malignancies are central, a biopsy sample is often obtained using fiber-optic bronchoscopy. A transthoracic biopsy or aspiration may be the first option if the tumor is peripheral [12]. The confirmatory diagnosis is histopathological.

Surgical resection with maximal preservation of the lung parenchyma is the treatment of choice in stages I and II CT and atypical carcinoid (CA). In the case of tumors of peripheral location, a segmentectomy or a lobectomy with regional lymphadenectomy should be performed [10-14]. Interventional bronchoscopy is reserved for patients with a high surgical risk. Local radiotherapy is indicated if the patient refuses surgery [14]. Adjuvant treatment, with a combination of radiation and chemotherapy, is utilized following surgery for AC and LCNEC patients, with demonstrable survival advantages [14]. Somatostatin analogs result in remission in up to 10% of symptomatic patients with hormone-active carcinoid tumors and in disease stability in 30-50% [15].

The prognosis mainly depends on the histological, biological, and clinical elements. Carcinoid tumors diagnosed with tumor, lymph node, and metastasis (TNM) stage I and II are operable with a five-year survival, unlike LCNECs and SCLCs which are often diagnosed late with a very low survival [12].

## Conclusions

NF1 represents an important risk factor to consider in the occurrence of even rare lung cancers which can be life-threatening for patient. Furthermore, pulmonary neuroendocrine tumors remain rare presenting with variable and sometimes misleading clinical pictures. Imaging examination is necessary for the establishment of diagnosis accompanied subsequently by a tissue biopsy. The treatment is controversial but the resection of the tumor remains the curative treatment especially in stages I and II of the TNM classification.
